# Successful Opioid Minimization Following Kidney Transplant: A Quality Improvement Initiative

**DOI:** 10.7759/cureus.52917

**Published:** 2024-01-25

**Authors:** Sarah Bova, Ron E Samet, Jacob Deering, Susanne Gaines, Abby Weinrub, Chandra Bhati, Silke Niederhaus

**Affiliations:** 1 Department of Pharmacy, University of Maryland Medical Center, Baltimore, USA; 2 Department of Anesthesiology, University of Maryland School of Medicine, Baltimore, USA; 3 Department of Pharmacy, University of Maryland School of Pharmacy, Baltimore, USA; 4 Department of Surgery, University of Maryland Medical Center, Baltimore, USA

**Keywords:** post-op pain management, transversus abdominis plane block (tap block), regional anesthesia, kidney transplantation, opioid analgesic

## Abstract

Opioid use after kidney transplant has been associated with an increased risk of death and graft loss. Several transplant centers have reported reductions in opioid use using multimodal analgesia and education. This study evaluated the impact of an opioid minimization protocol on inpatient opioid use and opioid prescribing on discharge.

This was a single-center, retrospective study of adult kidney recipients transplanted from October 2021 to July 2022. Patients on chronic opioids prior to transplant were excluded. The protocol included an intra-operative ultrasound-guided lateral transversus abdominis plane (TAP) block combined with scheduled non-opioid analgesics and tramadol as needed. Acetaminophen 1000 mg and gabapentin 300 mg were given 1 hour prior to the procedure and continued three times daily after transplant. The gabapentin dose was reduced for patients with renal impairment. Additional analgesics including opioids could be added for uncontrolled pain. We hypothesized the protocol would decrease total inpatient morphine milligram equivalents (MMEs) and opioid prescribing on discharge.

Fifty-nine post-protocol patients were compared to 52 pre-protocol patients. After the protocol, there was a significant decrease in total inpatient MMEs per day administered and no patient-controlled analgesia (PCA) devices were required. In alignment with the protocol, there was a significant increase in the use of TAP blocks, acetaminophen, gabapentin, and lidocaine patches. While opioid use was lowest in post-protocol patients who received TAP blocks, significant reductions in MMEs per day were still seen in those post-protocol who did not receive TAP blocks. Opioid prescribing at the time of discharge decreased significantly after protocol. No difference was seen in patient-reported pain scores, return to operating room, readmission within 30 days, or length of stay.

The use of scheduled acetaminophen and gabapentin with or without a TAP block allowed the elimination of PCA devices and led to significant minimizations in both inpatient opioid use and opioid prescribing on discharge.

## Introduction

Managing postoperative pain after a kidney transplant can be challenging and a strategy has not yet been established. Opioids are frequently relied upon for postoperative pain management; however, opioid use following a kidney transplant has been shown to increase the risk of morbidity and mortality [1]. There is now growing data to support opioid minimization and enhanced recovery after surgery (ERAS) protocols after kidney transplantation with safety and efficacy benefits [2,3]. Published opioid minimization protocols for kidney transplant recipients include an intraoperative transversus abdominis plane (TAP) block followed by scheduled acetaminophen and gabapentin, with opioids reserved for inpatients for breakthrough pain only and implementation of strict criteria for opioid prescribing on discharge [2]. In addition to optimizing analgesic use, ERAS protocols have also avoided prolonged preoperative fasting, encouraged early nutrition and mobilization, and removed invasive lines and catheters as early as possible. Multidisciplinary education and collaboration are essential for the implementation of these new protocols [4].

At our institution prior to the initiation of this protocol, kidney transplant recipients received intravenous hydromorphone either as needed or via patient-controlled analgesia (PCA) devices, then transitioned to as-needed oxycodone which was continued on discharge. Postoperative pain is managed by a multidisciplinary team consisting of transplant surgeons, anesthesiologists, pharmacists, nurse practitioners, nephrologists, and bedside nurses. This quality improvement protocol was developed to decrease inpatient opioid use and the use of prescription opioids on discharge. Other elements of ERAS including early mobilization and early nutrition were also included in hopes of decreasing hospital length of stay.

This article was previously presented as a meeting abstract at the 2023 American Transplant Congress on June 5, 2023. 

## Materials and methods

This retrospective cohort study compared outcomes before and after the implementation of an opioid minimization protocol. Adult kidney transplant patients transplanted between October 2021 and February 2022 were included in the pre-protocol group and patients transplanted between March 2022 and July 2022 were included in the opioid minimization group. Patients with a history of chronic opioid use, defined as multiple opioid prescription fills in the three months prior to transplant, were excluded. Patients receiving multi-organ transplants, including simultaneous pancreas-kidney and liver-kidney transplants, were also excluded.

**Figure 1:**
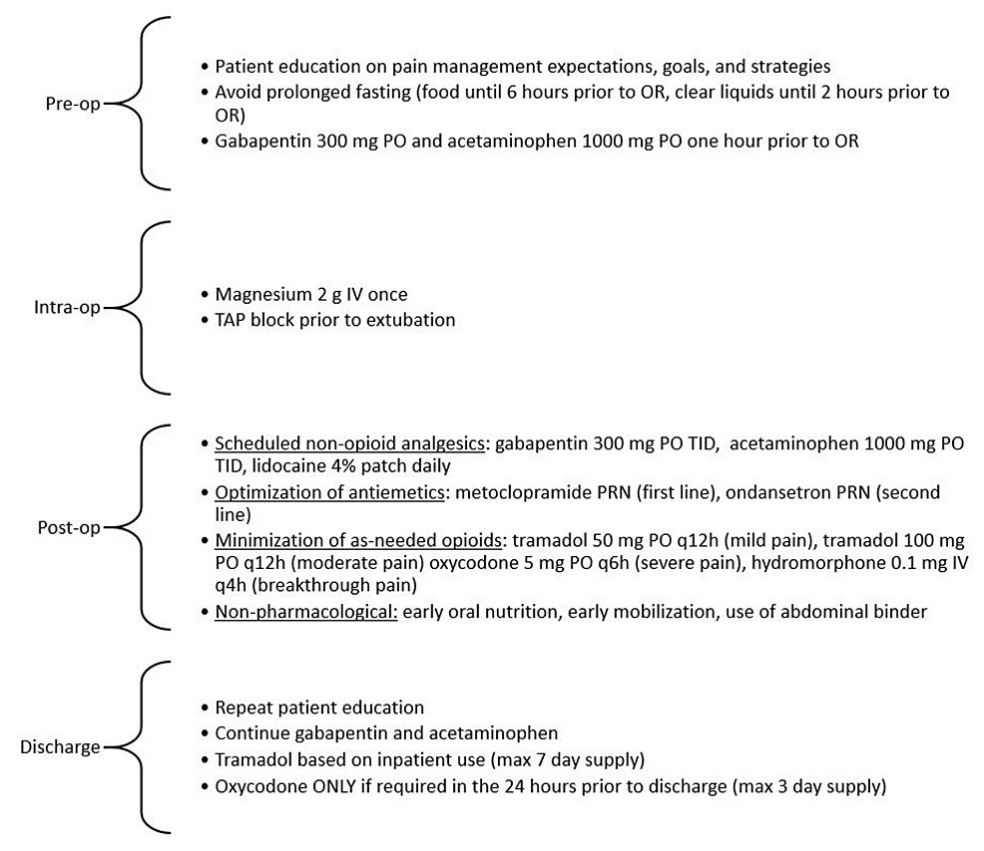
Opioid Minimization Protocol

The protocol stressed patient and staff education, administration of an intraoperative TAP block, and scheduled postoperative non-opioid analgesics (Figure 1). Patients received education from transplant surgeons, anesthesiologists, and bedside nurses as well as written materials on what to expect after transplant. Bupivacaine or ropivacaine (20-60 mL of 2.5 mg/mL) was used for lateral TAP blocks based on provider preference and the need for one-sided or bilateral blocks. Liposomal bupivacaine was not used due to hospital formulary restrictions and higher costs. When the regional anesthesia team was not available to perform TAP blocks, some transplant surgeons performed wound infiltration with 0.25% bupivacaine prior to abdominal closure. All patients were automatically ordered protocol medications via a postoperative order set in the electronic health record (EHR). Acetaminophen (1000 mg PO TID) and gabapentin (300 mg PO TID) were started preoperatively and continued through discharge. Gabapentin dosing was adjusted based on renal function (300 mg PO daily for CrCl <30 mL/min, 300 mg PO BID for CrCl 30-50 mL/min, and 300 mg PO TID for CrCl >50 mL/min). The transplant team was able to change analgesic orders as needed if patients were experiencing uncontrolled pain. Patients were expected to be out of bed to a chair on postoperative day (POD) 1, and ambulating by POD2. Diet was advanced to clear liquid then solid food as tolerated by patients. All patients received a steroid taper as part of their immunosuppression per center protocol (methylprednisolone 500 mg IV in OR, 250 mg IV on POD1, 125 mg IV on POD2, followed by a 21-day oral prednisone taper). On discharge, providers followed strict criteria for prescribing tramadol and oxycodone for as-needed analgesics (Figure 1).

The primary outcomes were inpatient opioid requirements on postoperative days 1 through 3, reported as total morphine milligram equivalents (MMEs) per day, and opioid prescribing on discharge. Secondary outcomes included non-opioid analgesic administration, use of PCA devices, patient-reported pain scores, return to operating room, length of stay, and readmission rate. Pain scores were assessed by bedside nurses using a numerical rating scale of zero to 10. Data were collected from the EHR. The study was reviewed by the local Institutional Review Board and determined to be exempt. Categorical variables were compared using the chi-square test and continuous variables were compared using the Wilcoxon rank-sum given the small sample size. For all analyses, p-value <0.05 was considered statistically significant. Analysis was performed using R Studio Statistical Software (RStudio Team (2021). Rstudio: Integrated Development Environment for R. Rstudio, PBC, Boston, MA).

## Results

A total of 111 patients were included in the study. There were no significant differences in demographics between the two groups at baseline (Table 1). There was a significant decrease in inpatient opioid use as well as opioid prescribing upon discharge in the post-protocol group (Table 2). Total MMEs per day on POD 1, 2, and 3 decreased from a median of 32, 15, and 0 before protocol to 2, 0, and 0 after protocol, respectively (p ≤0.005, 0.005, 0.04). On discharge, there was a significant decrease in the number of patients prescribed oxycodone and an increase in patients prescribed tramadol in accordance with the protocol. Regardless of the agent prescribed, there was a significant decrease in the total quantity of opioid tablets prescribed after discharge, from 30 before protocol to 14 after protocol.

**Table 1:** Baseline Characteristics

Characteristic	Pre-protocol (N=52)	Post-protocol (N=59)	p-Value
Age at transplant (years), mean ± SD	56.8 ± 14	53.2 ± 12.5	0.17
Male gender, n (%)	36 (69.2)	32 (54.2)	0.118
Race, n (%)			
White	20 (38.5)	17 (28.8)	0.323
Black	29 (55.8)	39 (66.1)	
Hispanic	2 (3.8)	1 (1.7)	
Asian	0 (0)	2 (3.4)	
Donor type, n (%)			
Deceased	41 (78.8)	41 (69.5)	0.399
Living	11 (21.2)	18 (30.5)	
BMI at transplant (kg/m^2^), median (IQR)	27.2 (24.3-32.5)	28.6 (24.1-34.3)	0.557

**Table 2:** Opioid Use After Transplant MME, morphine milligram equivalent; POD, postoperative day.

Characteristic	Pre-protocol (N=52)	Post-protocol (N=59)	p-Value
Total MME per day, median (IQR)			
POD1	32 (13-81)	2 (0-22.5)	<0.005
POD2	15 (0-40.8)	0 (0-7.5)	0.005
POD3	0 (0-17.5)	0 (0-7.5)	0.04
Analgesic at discharge, n (%)			
Oxycodone 5 mg	33 (63.5)	19 (32.2)	<0.005
Tramadol 50 mg	9 (17.3)	28 (47.5)	
Analgesic quantity at discharge, median (IQR)	30 (20-30)	14 (12-20)	<0.005

PCA devices were the main pain management strategy pre-protocol with 71.2% of patients prescribed one. PCA use was eliminated with no patients requiring a PCA after protocol (Table 3). In place of a PCA, there was a significant increase in TAP blocks from pre- to post-protocol (17.3% to 47.5%, p≤0.005). Thirty-one post-protocol patients were not able to receive TAP blocks due to the timing of the transplant and the availability of the regional anesthesia team. Seven of these patients received local anesthetic infiltration by the surgical team prior to closure. When comparing pre-protocol patients with post-protocol patients who did not receive TAP blocks (n=31), there was still a significant decrease in inpatient MMEs for the first three days postoperatively (59.0 vs 22.5, p=0.009). An even greater decrease in MMEs was appreciated in post-protocol patients who received TAP blocks (59.0 vs 0, p<0.005). There were significant increases in non-opioid analgesic use with nearly all patients receiving lidocaine patches, gabapentin, and acetaminophen after protocol (p<0.005). Tramadol use did increase after protocol; however, the median total daily dose on POD 2 and 3 was 0 in both groups. There were no differences in patient-reported pain scores, return to OR, length of stay, or readmission rates.

**Table 3:** Secondary Outcomes TAP, transversus abdominis plane; PCA, patient-controlled analgesia; POD, postoperative day.

Characteristic	Pre-protocol (N=52)	Post-protocol (N=59)	p-Value
TAP block, n (%)	9 (17.3)	28 (47.5)	0.002
Intraoperative lidocaine infusion, n (%)	2 (3.8)	0 (0)	0.412
Intraoperative ketamine infusion, n (%)	5 (9.6)	1 (1.7)	0.148
Intraoperative wound infiltration with bupivacaine, n (%)	10 (19.2)	7 (11.9)	0.392
PCA, n (%)	37 (71.2)	0 (0)	<0.005
Lidocaine patch, n (%)	19 (36.5)	57 (96.6)	<0.005
Gabapentin, n (%)	17 (32.7)	58 (98.3)	<0.005
Acetaminophen (TDD in mg), median (IQR)			
POD1	1000 (0-2825)	3000 (2000-3000)	<0.005
POD2	1300 (0-3000)	3000 (2325-3000)	<0.005
POD3	1000 (0-2450)	2000 (2000-3000)	<0.005
Tramadol (TDD in mg), median (IQR)			
POD1	0 (0-0)	50 (0-100)	<0.005
POD2	0 (0-0)	0 (0-50)	0.02
POD3	0 (0-0)	0 (0-0)	0.022
Pain scores, median (IQR)			
POD1	7 (4.5-8.5)	7 (5-8)	0.714
POD2	6 (3.5-8)	5 (3-7.5)	0.427
POD3	4.5 (2-6.8)	4 (2-7)	0.536
Return to OR, n (%)	3 (5.8)	4 (6.8)	1
Length of stay (days), median (IQR)	4 (3-6)	4 (3-6)	0.225
Readmission within 30 days, n (%)	12 (23.1)	20 (33.9)	0.325

## Discussion

There are growing data to support the feasibility, efficacy, and importance of opioid minimization following kidney transplant. The purpose of this study was to evaluate the degree of impact implementation of an opioid minimization protocol can have in our kidney transplant population. Administration of a TAP block or other local anesthetic modality in combination with scheduled acetaminophen, gabapentin, and lidocaine patches allowed for as-needed only tramadol use and elimination of PCA use in our population. This multimodal protocol resulted in significantly decreased inpatient opioid use as well as a dramatic decrease in opioid prescribing upon discharge. Patients receiving a TAP block performed by the regional anesthesia team had a larger decrease in postoperative opioid requirements compared to patients who did not receive a TAP block, though benefits of multimodal management were seen in both groups. Multidisciplinary staff and patient education along with the creation of a standard order set in the EHR were key to successful protocol implementation.

Gopwani and Rosenblatt showed that the use of a TAP block with 0.25% bupivacaine significantly reduced opioid requirements at six, 12, 24, and 48 hours following kidney transplant [5]. Others have combined this intervention with scheduled postoperative acetaminophen and gabapentin with positive results [6,7]. Rohan et al showed reductions in both inpatient opioid requirements and opioid prescribing on discharge with a decrease from 96% to just 5% of kidney recipients being discharged with an opioid prescription [6]. Muir et al. added intraoperative ketamine in addition to preoperative pregabalin, a TAP block, and scheduled postoperative APAP and gabapentin [7]. They showed reductions in inpatient opioid use, readmissions, and outpatient opioid use within six months of discharge. Given their success with minimal to no adverse effects, our protocol interventions were developed based upon these studies. Like Gopwani and Rosenblatt and Rohan et al., the TAP blocks were performed by our regional anesthesia team or the anesthesiologist at the end of the operation. Due to availability, less than half of our post-protocol patients received a TAP block. Despite significant reductions in opioid requirements in the post-protocol population as a whole, we observed even further reductions in opioid requirements for those who received a TAP block compared to those who did not. When ultrasound-guided TAP blocks are unavailable, surgical local anesthetic infiltration can potentially provide nociceptive analgesia as well, and is recommended.

While TAP blocks were the main component of our protocol, it is important to adequately appreciate the value of standardized enteral and topical non-opioid analgesics. Schwab et al. studied a protocol of scheduled acetaminophen, gabapentin, and lidocaine patches without a TAP block and found significant reductions in PCA use from 81% to 4.2% [8]. These findings align with our results as patients who did not receive a TAP block still had lower opioid requirements compared to pre-protocol patients. A systematic review by Kutzler and colleagues recommends incorporation of scheduled acetaminophen and gabapentin into pain management protocols for kidney transplant recipients [2]. Anecdotally, post-protocol patients reported improved analgesia from lidocaine patches and many continued using them after discharge.

Efforts to minimize opioid use should continue upon discharge. To facilitate this effort, clear direction should be provided to prescribers. Schwab et al. implemented an opioid minimization protocol, which included several inpatient interventions but no guidance for discharge prescribing [8]. They found significant decreases in opioid PCA use but no difference in opioid prescribing upon discharge. They subsequently added guidelines specific to minimizing opioid prescribing upon discharge and saw a significant decrease in opioid prescribing [9]. Our protocol also included specific guidance for discharge prescribing of tramadol versus oxycodone, including strengths and quantities. Inclusion of this guidance led to reduced oxycodone prescribing and reduced numbers of tablets prescribed for either analgesic.

Our study has several limitations. It is a single-center study and may not be applicable elsewhere as patient populations may differ. Sample size calculations were not performed as our protocol began as a quality-improvement initiative. While we hoped for better adherence to the administration of TAP blocks for the majority of the post-protocol patients, the regional anesthesia team was unavailable during nights and weekends limiting this intervention. The creation of an order set helped ensure all patients were ordered the protocol medications; however, the transplant team was able to modify the orders as clinically indicated. We also only studied opioid usage up until hospital discharge and opioid prescriptions upon discharge, but we did not collect data of opioid use or refills after discharge. Our control group is historic; though the difference in time only spans months, there may have been differences in stress levels of patients which may have contributed to different perceptions of pain. Lead-time bias is unlikely as this study did not include long-term follow-up. Finally, we excluded patients with a history of chronic opioid use prior to transplant limiting our findings' applicability to that cohort.

Strengths include a diverse patient population, and the finding that even if adherence to the protocol is not always feasible, which will be a realistic challenge for other centers as well, there is still significant benefit even when TAP blocks are unavailable. The clarity of the inpatient and discharge opioid prescribing guidelines would be easily transferrable to most other centers looking to implement changes.

## Conclusions

In conclusion, our opioid minimization protocol was successful in reducing inpatient opioid requirements and opioid prescribing upon discharge in kidney transplant recipients. Furthermore, we were able to eliminate the use of PCAs completely in our post-protocol patients. Not surprisingly, there was a significant increase in the use of non-opioid analgesics including acetaminophen, gabapentin, and local anesthetics, favoring a multimodal approach to successful analgesia in kidney transplant recipients. Future research is recommended to determine the impact of opioid minimization protocols on transplant recipients of other organs, as well as for chronic opioid users.
